# Supplementary material for: An estimate of absolute shear-wave speed in the Earth’s inner core
Source: Nat Commun. 2023 Jul 29;14:4577. doi: 10.1038/s41467-023-40307-9 (PMC10387060; doi:10.1038/s41467-023-40307-9)
Supplement: Supplementary file 1 — Supplementary Information [file 41467_2023_40307_MOESM1_ESM.pdf]

Supplementary Information

An estimate of absolute shear-wave speed in the Earth's inner core

Thuany Costa de Lima[1,*], Thanh-Son Phạm[1], Xiaolong Ma[1], and Hrvoje Tkalčić[1]

[1]Research School of Earth Sciences, The Australian National University, Canberra, ACT, Australia

* Corresponding author: (Thuany.CostadeLima@anu.edu.au)

Content of this file

Figures S1 to S4

Table S1 and S2

Supplementary Movie 1

Supplementary Figures

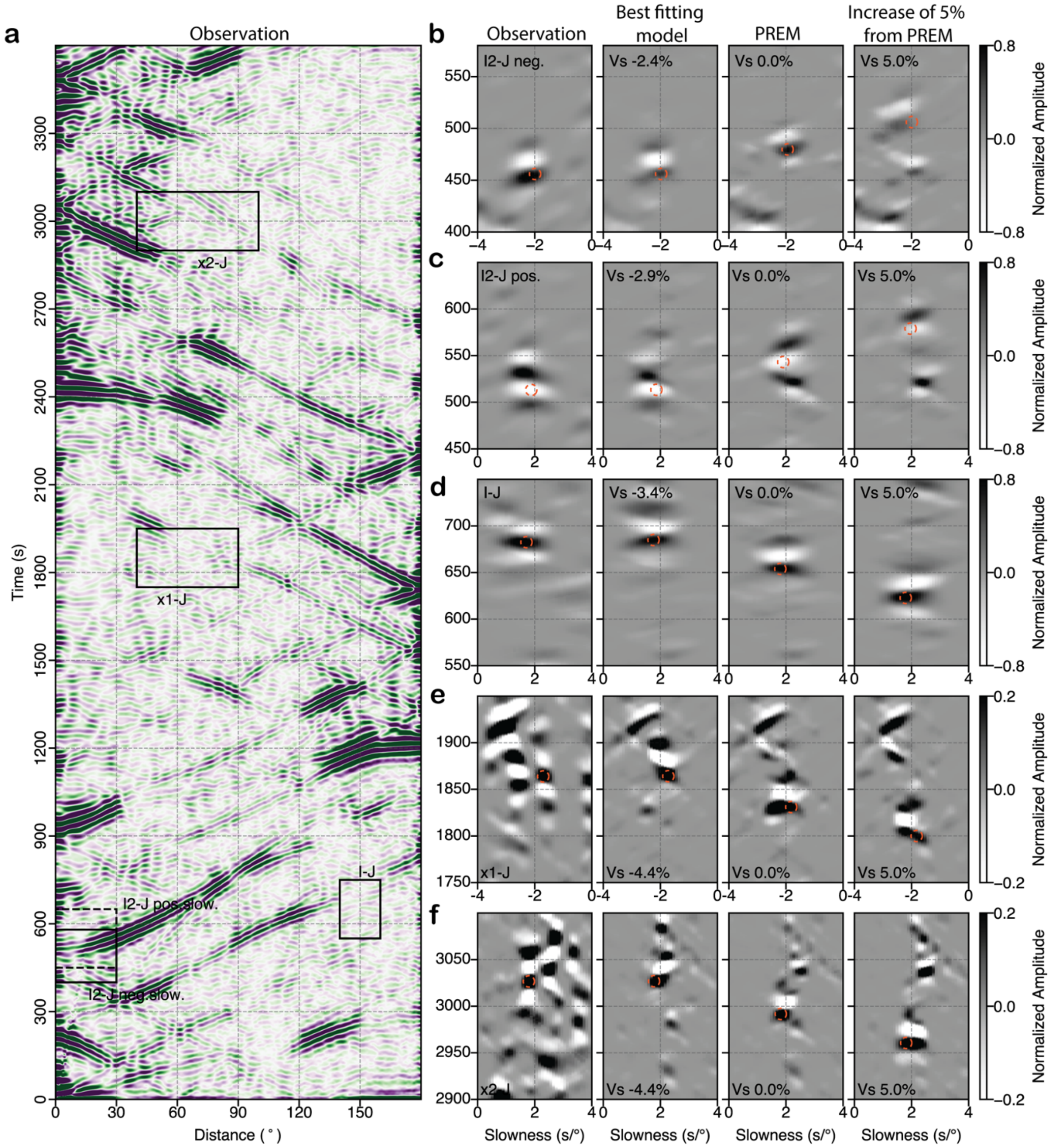

Figure S1. Systematic detection of J-waves in the global correlogram. (**a**) Observed correlogram after data selection (see Methods). The rectangles in black indicate the time and distance windows of the correlation features sensitive to the IC shear-wave speed identified via numerical experiments (Animation S1). (**b-f**) Phase-weighted slant stacks of correlation waveforms calculated within the inset boxes in panel (**a**). Columns from the left to the right in (**b-f**) are the slant stack of observation, synthetics calculated for the best fitting model, PREM (0% change), and model with an increase of 5% in the IC shear wave velocity from PREM, respectively. The latter two panels are shown for reference purposes. The red circles in (**b-f**)

indicate the arrival times of I2-J negative slowness leg (**b**), I2-J positive slowness leg (**c**), I-J (**d**), x1-J (**e**), and x2-J (**f**).

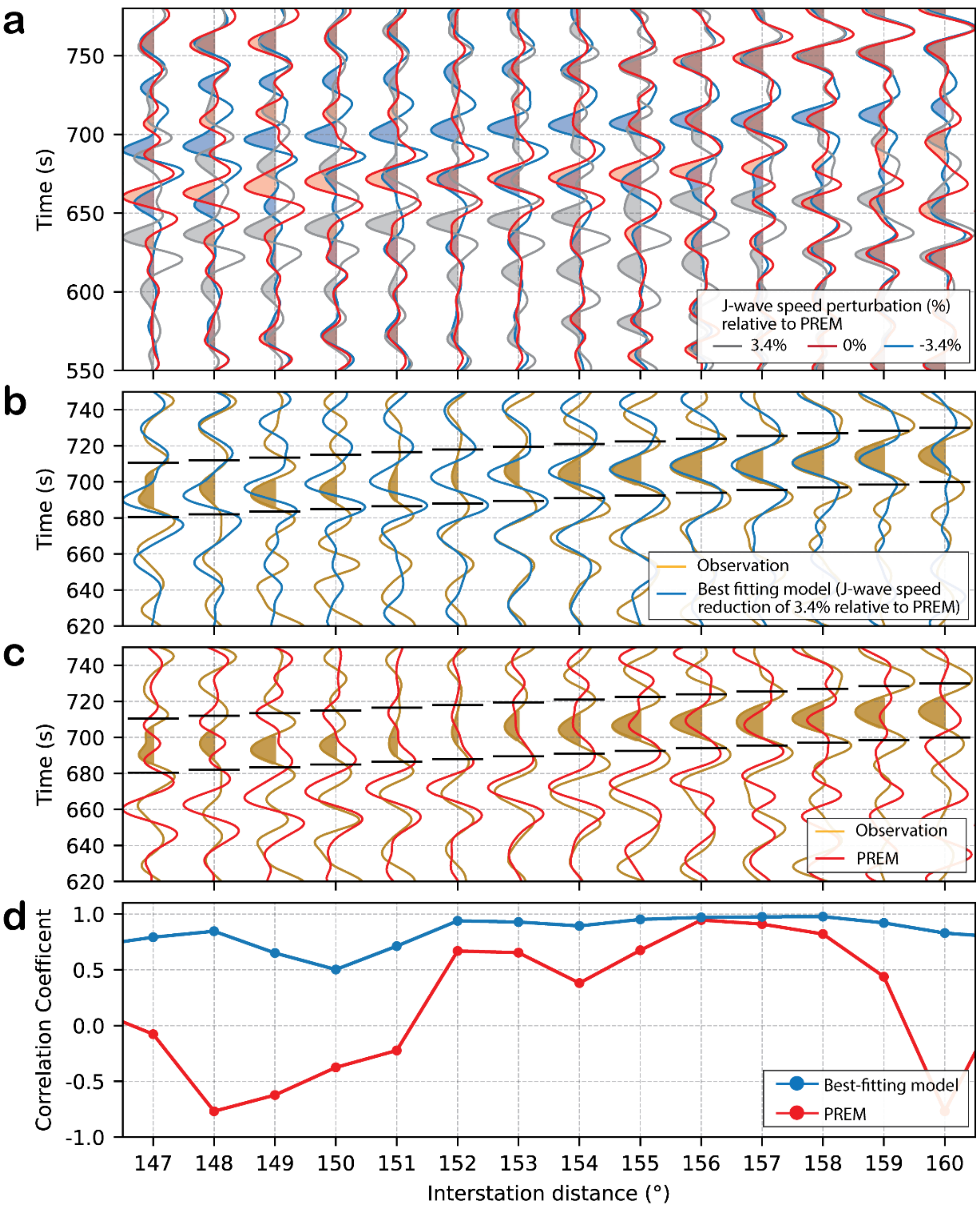

Figure S2. Sensitivity of feature I-J to the shear-wave speed in the IC without slant-stacking. (**a**) Timing variation of synthetic waveforms of feature I-J for different J-wave speeds relative to PREM: reduction of 4.5%, no perturbation from PREM (0% change), and an increase of 4.5% (waveforms in red, grey, and blue, respectively) at T=23.1s. Each correlation waveform corresponds a vertical strip shown in the 2D correlogram, for instance, shown in Fig. S1a. (**b**) Observation of feature I-J (waveforms in dark yellow), and best fitting model estimated with a reduction of 3.4% in J-wave speed relative to PREM (waveforms in blue) at

T=1s. The arrival times of I-J are highlighted by pairs of horizontal solid black lines at each interstation distance (note that this panel is a repetition of Fig. 4b from the main text). (**c**) Similar to **b**, but the synthetic model is based on PREM (waveforms in red). (**d**) Correlation coefficients (CC) estimated as a function of interstation distances between observed and synthetic waveforms of panels (b) and (c) are plotted in blue and red, respectively. The CC values are calculated for waveforms bounded by horizontal solid black lines at each interstation distance.

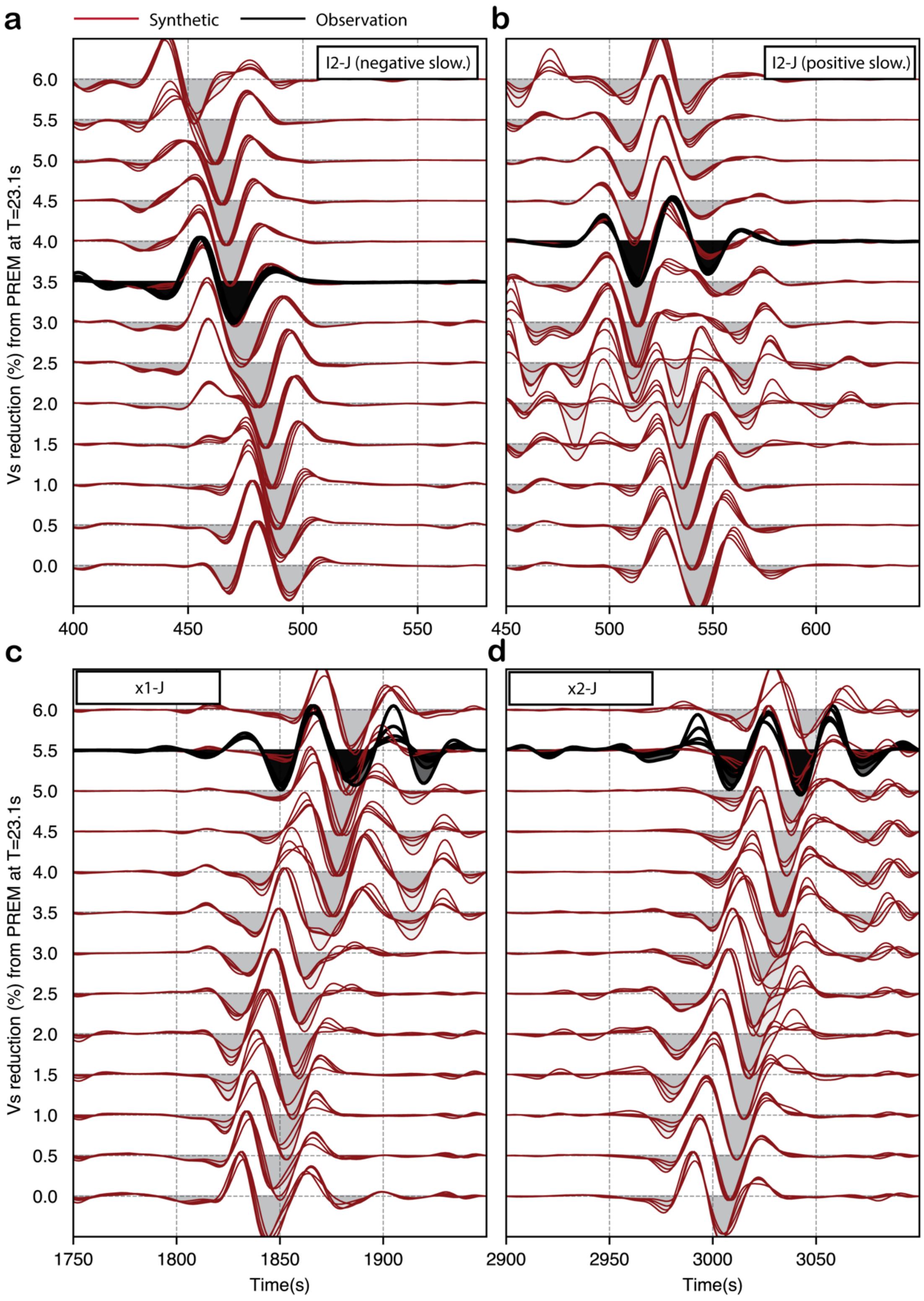

Figure S3. Best fitting of J-wave speeds for four correlation features (apart from feature I-J). (**a**) Slant stacks of the negative slowness leg of I2-J at the slowness of -1.5, -1.6, -1.7, -1.8, and -1.9 s/° estimated for models with different J-wave perturbations from PREM sorted in the vertical axis. The observed waveforms are in black, and the synthetic waveforms are in red. (**b-d**) similar to (**a**) but for the positive slowness leg of I2-J, x1-J, and x2-J features,

respectively. The best-fitting reduction values to fit each of the correlation features is chosen based on the correlation coefficient (CC) and documented in Table S1.

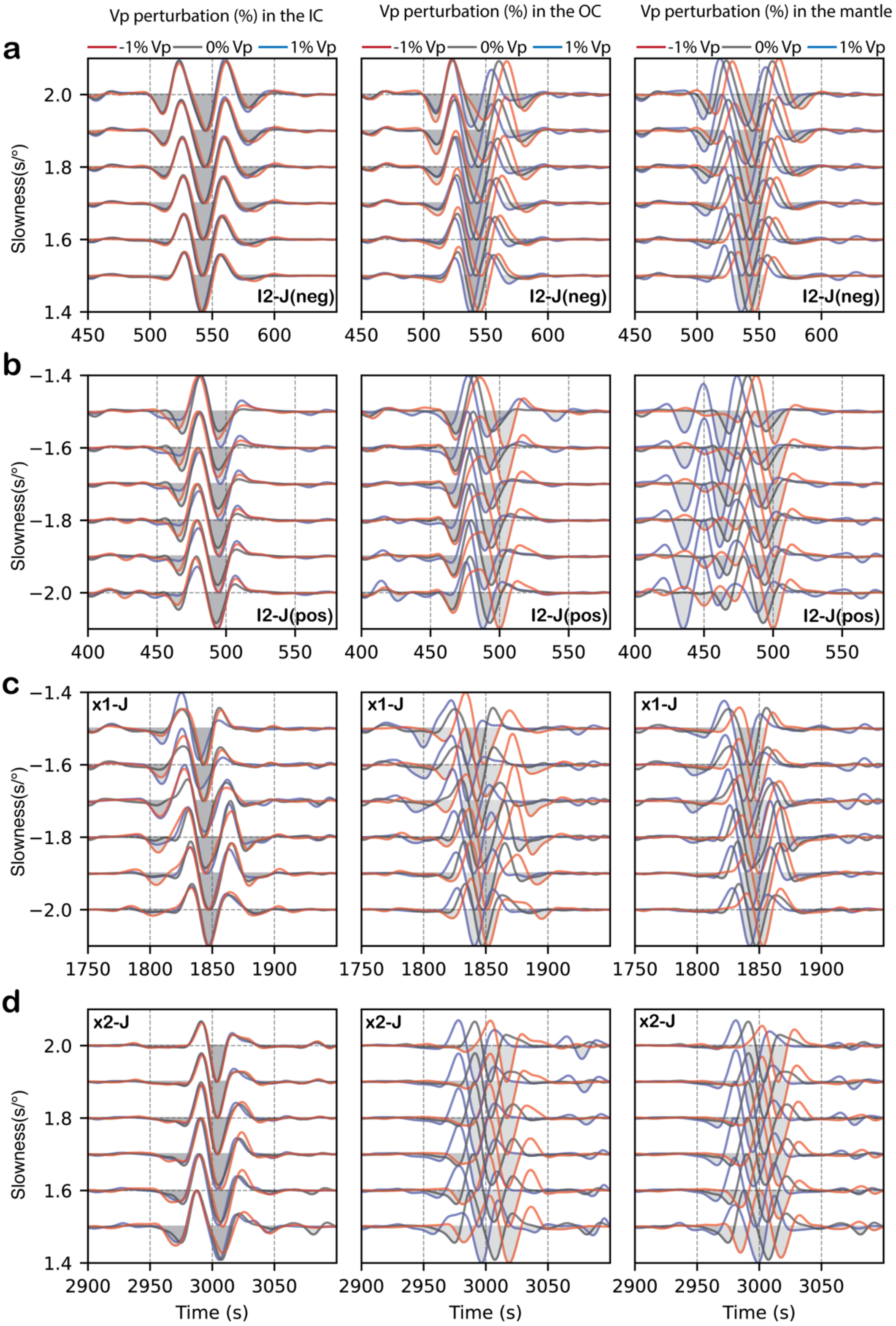

Figure S4. Sensitivity of J-wave correlation features of compressional wave speeds in the mantle, outer core, and inner core. (**a-d**) Slant stacks of I2-J (negative slowness leg), I2-J (positive slowness leg), x1-J, and x2-J, respectively, calculated for a range of slowness sorted in the vertical axis. The slant stacks are computed for models with perturbations in

compressional wave velocity profile in the inner core (left panel), outer core (middle panel), and mantle (right panel) of +1%, 0%, and -1%, in blue, grey, and red waveforms, respectively.

Table S1. Correlation coefficient between observation and the synthetic counterpart of correlation features calculated for a range of J-wave velocity perturbation from PREM, and CCREM. The time and distance windows used to calculate the slant stacks are indicated in the header of the table. The best-fitting model of each correlation feature is chosen based on the highest correlation coefficient (highlighted in grey).

**Correlation Feature**	I2-J (positive slowness leg)	I-J	x2-J	I2-J (negative slowness leg)	x1-J
**Time window (s)**	450-650	550-750	2900-3100	400-580	1750-1950
**Angular distance window (°)**	0-30	140-160	40-100	0-30	40-90
**Vs reduction (%) from PREM at T=1s using the central period of T=23.1s for correction**	**Correlation Coefficient** (Median from synthetic and observed slant stacks at the slowness range from 1.5 to 2.0)			**Correlation Coefficient** (Median from synthetic and observed slant stacks at the slowness range from -2.0 to -1.5)	
-1.2	0.42	0.58	0.45	0.03	0.62
-0.7	0.14	0.22	0.54	-0.26	0.64
-0.2	-0.20	-0.43	0.45	-0.42	0.55
0.3	-0.38	-0.86	0.17	-0.28	0.26
0.8	-0.42	-0.86	-0.22	0.07	-0.19
1.3	0.14	-0.55	-0.45	0.52	-0.52
1.8	0.56	-0.02	-0.55	0.81	-0.72
2.3	0.80	0.48	-0.40	0.94	-0.63
2.9	0.92	0.77	-0.01	0.85	-0.17
3.4	0.86	0.81	0.44	0.56	0.35
3.9	0.67	0.54	0.71	0.18	0.65
4.4	0.42	0.07	0.80	-0.37	0.76
4.9	0.19	-0.32	0.62	-0.72	0.68
**Vs reduction (%) from CCREM**					
0.0	0.72	0.70	0.58	0.37	0.57
0.5	0.57	0.50	0.53	0.33	0.78
1.0	0.36	0.05	0.40	0.22	0.76
1.5	0.13	-0.55	0.07	-0.12	0.48
2.0	-0.12	-0.88	-0.40	-0.19	-0.01
2.5	-0.05	-0.82	-0.57	-0.04	-0.39
3.0	0.36	-0.42	-0.61	0.26	-0.71
3.5	0.62	0.16	-0.22	0.59	-0.74
4.0	0.87	0.63	0.31	0.84	-0.28
4.5	0.95	0.85	0.60	0.88	0.35
5.0	0.88	0.75	0.83	0.72	0.67
5.5	0.75	0.36	0.81	0.38	0.79
6.0	0.54	-0.10	0.37	-0.11	0.78

Table S2. Origin time, depth, latitude, and longitude of earthquakes that we use in this study as listed in the GCMT catalog.

Origin time	latitude	longitude	depth	magnitude
2010-01-03T22:36:42.400000Z	157.21	-8.88	12.0	7.11
2010-01-05T12:15:45.500000Z	157.77	-9.11	12.0	6.83
2010-02-18T01:13:23.200000Z	130.66	42.48	578.7	6.88
2010-02-27T08:01:29.800000Z	-75.41	-38.09	19.9	7.36
2010-03-11T14:55:35.600000Z	-72.13	-34.53	16.3	6.96
2010-04-06T22:15:19.100000Z	96.74	2.07	17.6	7.81
2010-04-11T09:40:33.900000Z	161.18	-11.04	36.1	6.85
2010-05-09T05:59:51.400000Z	95.78	3.36	37.2	7.25
2010-05-27T17:14:55.400000Z	166.65	-13.81	42.9	7.16
2010-07-18T13:04:16.100000Z	150.68	-6.32	35.0	6.92
2010-07-18T13:35:13.100000Z	150.33	-6.22	36.7	7.32
2010-07-23T22:51:20.700000Z	123.9	6.62	576.9	7.64
2010-07-23T23:15:15.700000Z	123.48	6.83	641.4	7.45
2010-08-04T22:01:55.100000Z	150.8	-6.26	34.8	6.89
2010-08-12T11:54:20.700000Z	-77.51	-1.51	197.8	7.07
2010-08-13T21:19:43.300000Z	141.52	12.46	12.0	6.89
2010-09-29T17:11:32.000000Z	133.78	-4.92	17.7	6.96
2010-10-25T14:42:59.800000Z	99.32	-3.71	12.0	7.82
2011-01-01T09:57:03.800000Z	-63.21	-27.02	586.0	7.03
2011-01-02T20:20:26.600000Z	-73.84	-38.71	19.4	7.13
2011-01-13T16:16:51.200000Z	168.34	-20.68	17.2	6.93
2011-01-18T20:23:31.800000Z	63.9	28.61	52.3	7.23
2011-02-11T20:05:39.100000Z	-73.56	-36.63	17.8	6.84
2011-03-09T02:45:32.000000Z	142.78	38.56	14.1	7.33
2011-03-11T06:15:58.700000Z	141.38	35.92	29.0	7.89
2011-03-11T06:26:12.600000Z	144.63	38.27	21.1	7.59
2011-04-07T14:32:50.600000Z	141.85	38.32	53.3	7.11
2011-06-24T03:09:51.500000Z	-171.77	52.09	74.2	7.25
2011-08-20T16:55:13.100000Z	167.94	-18.52	33.5	7.13
2011-08-20T18:19:35.400000Z	167.94	-18.26	36.0	7.04
2011-08-30T06:57:47.700000Z	126.68	-6.47	468.7	6.88
2011-10-21T17:57:28.300000Z	-175.72	-28.83	48.4	7.38
2011-10-28T18:54:43.100000Z	-76.12	-14.52	25.1	6.95
2012-01-01T05:28:01.400000Z	138.17	31.61	352.0	6.79
2012-03-14T09:08:42.800000Z	144.93	40.88	13.0	6.95
2012-03-20T18:02:54.900000Z	-98.39	16.6	15.4	7.46
2012-03-25T22:37:20.900000Z	-72.41	-35.31	33.8	7.12
2012-08-14T02:59:50.900000Z	145.7	49.97	598.2	7.72
2012-08-27T04:37:39.500000Z	-89.17	12.02	12.0	7.34
2012-09-05T14:42:23.300000Z	-85.64	10.0	29.7	7.62
2014-10-14T03:51:43.700000Z	-88.45	12.33	40.8	7.28
2014-11-01T18:57:29.900000Z	-177.61	-19.76	445.4	7.11
2014-11-15T02:31:49.800000Z	126.37	1.98	38.1	7.05
2014-11-26T14:33:50.000000Z	126.44	2.11	35.2	6.77
2015-03-29T23:48:54.700000Z	152.59	-5.18	37.6	7.45
2015-04-25T06:11:58.600000Z	85.33	27.91	12.0	7.88
2015-05-05T01:44:26.600000Z	152.1	-5.32	38.3	7.46
2015-05-07T07:10:33.600000Z	154.49	-7.36	12.0	6.98
2015-05-12T07:05:27.500000Z	86.08	27.67	12.0	7.23
2015-05-12T21:13:06.100000Z	142.29	38.89	43.9	6.81
2015-05-30T11:23:11.000000Z	140.56	27.94	680.7	7.86
2015-07-18T02:27:40.000000Z	165.1	-10.35	12.3	6.93
2015-07-27T04:49:52.600000Z	-169.42	52.21	27.1	6.9
2015-09-16T22:55:22.900000Z	-72.09	-31.13	17.4	8.27
2015-09-16T23:18:52.100000Z	-71.95	-31.79	35.7	7.1
2015-09-17T04:10:36.300000Z	-71.94	-31.65	36.6	6.76
2015-10-26T09:09:46.600000Z	70.42	36.55	209.4	7.5
2015-11-11T02:46:33.000000Z	-72.29	-29.7	12.0	6.79
2015-11-18T18:31:12.400000Z	158.44	-9.11	21.5	6.84
2015-11-24T22:51:00.700000Z	-71.28	-10.11	627.3	7.65
2015-12-09T10:21:54.600000Z	129.51	-4.16	12.2	6.79
2016-04-03T08:23:59.100000Z	166.66	-14.27	34.8	6.82
2016-04-16T23:58:57.000000Z	-80.25	-0.12	22.3	7.78
2016-04-28T19:33:33.700000Z	167.26	-16.05	33.5	6.97
2016-05-18T16:46:49.200000Z	-80.02	0.3	33.6	6.87
2016-05-28T05:38:57.400000Z	-178.17	-22.13	417.5	6.92
2016-08-19T07:32:38.400000Z	-31.41	-55.16	22.1	7.46
2016-08-24T10:34:58.300000Z	94.58	20.79	88.3	6.76
2016-08-31T03:11:40.600000Z	152.86	-3.58	491.0	6.78
2016-09-01T16:38:15.800000Z	179.03	-37.19	27.8	7.07
2016-09-24T21:28:48.400000Z	-178.15	-19.77	606.8	6.92
2016-11-21T20:59:58.200000Z	141.46	37.31	12.0	6.93
2016-11-24T18:43:53.600000Z	-89.2	11.83	12.0	6.93
2016-12-09T19:10:26.900000Z	161.06	-10.95	12.0	6.91
2016-12-17T10:51:56.300000Z	153.76	-5.55	52.8	7.88
2016-12-25T14:22:38.000000Z	-74.43	-43.41	32.8	7.57
2017-01-03T21:52:38.400000Z	176.0	-19.28	17.3	6.93
2017-01-10T06:13:55.900000Z	122.78	4.57	621.5	7.27
2017-01-22T04:30:38.100000Z	154.94	-6.03	149.6	7.89
2017-02-24T17:28:56.700000Z	-178.77	-23.44	417.9	6.95

2012-09-30T16:31:44.500000Z | -76.22 | 1.89 | 159.1 | 7.24
2012-10-28T03:04:37.200000Z | -132.06 | 52.61 | 12.0 | 7.77
2012-11-07T16:35:56.300000Z | -92.43 | 14.11 | 21.3 | 7.37
2012-12-07T08:18:46.900000Z | 143.83 | 37.77 | 19.5 | 7.19
2013-02-06T01:12:55.000000Z | 165.21 | -11.18 | 20.2 | 7.91
2013-02-06T01:23:30.200000Z | 164.86 | -11.32 | 22.5 | 7.09
2013-04-16T10:44:32.200000Z | 62.21 | 27.89 | 50.8 | 7.74
2013-04-19T03:06:04.700000Z | 150.92 | 46.0 | 104.5 | 7.25
2013-05-23T17:19:13.000000Z | -176.88 | -23.19 | 188.1 | 7.41
2013-05-24T05:45:08.400000Z | 153.77 | 54.61 | 611.0 | 8.33
2013-07-07T18:35:42.400000Z | 153.98 | -4.1 | 382.9 | 7.3
2013-08-30T16:25:09.500000Z | -175.12 | 51.44 | 26.7 | 6.97
2013-09-25T16:42:52.400000Z | -74.81 | -16.03 | 46.1 | 7.04
2013-10-12T13:11:56.400000Z | 23.37 | 35.37 | 15.0 | 6.76
2013-10-15T00:12:50.000000Z | 123.96 | 9.84 | 12.0 | 7.1
2013-10-16T10:31:05.200000Z | 154.93 | -6.69 | 45.8 | 6.78
2013-10-25T17:10:25.300000Z | 144.66 | 37.17 | 24.9 | 7.14
2014-04-01T23:58:11.400000Z | -71.39 | -19.85 | 20.6 | 7.0
2014-04-11T07:07:33.900000Z | 155.09 | -6.78 | 44.1 | 7.07
2014-04-13T12:36:28.100000Z | 161.97 | -11.45 | 37.5 | 7.44
2014-04-18T14:27:36.100000Z | -101.25 | 17.55 | 18.9 | 7.27
2014-04-19T13:28:12.800000Z | 154.67 | -6.64 | 36.0 | 7.51
2014-06-23T19:19:25.300000Z | -177.13 | -30.03 | 26.4 | 6.88
2014-06-29T07:53:05.700000Z | -28.31 | -55.34 | 21.0 | 6.85
2014-07-07T11:23:59.400000Z | -92.7 | 14.8 | 62.9 | 6.9
2014-07-21T14:54:46.900000Z | -178.32 | -19.68 | 627.1 | 6.91
2014-08-24T23:21:49.700000Z | -73.72 | -14.61 | 84.5 | 6.83
2014-09-17T06:14:49.900000Z | 144.51 | 13.54 | 140.4 | 6.75
2014-10-09T02:14:42.400000Z | -110.81 | -32.34 | 12.0 | 6.95
2017-04-24T21:38:37.400000Z | -72.07 | -33.13 | 26.0 | 6.86
2017-04-28T20:23:23.600000Z | 124.89 | 5.49 | 31.4 | 6.85
2017-06-14T07:29:07.900000Z | -92.17 | 14.92 | 72.7 | 6.95
2017-06-22T12:31:09.100000Z | -91.38 | 13.57 | 38.1 | 6.77
2017-09-08T04:49:46.700000Z | -94.66 | 15.38 | 44.8 | 8.23
2017-09-19T18:14:47.200000Z | -98.63 | 18.59 | 51.0 | 7.14
2017-11-12T18:18:25.300000Z | 45.84 | 34.83 | 17.9 | 7.4
2017-11-19T22:43:43.200000Z | 168.67 | -21.45 | 12.0 | 6.96
2018-01-14T09:18:52.300000Z | -74.78 | -15.95 | 40.9 | 7.12
2018-02-16T23:39:46.300000Z | -97.88 | 16.53 | 20.0 | 7.16
2018-02-25T17:45:08.600000Z | 142.97 | -6.29 | 12.0 | 7.47
2018-03-29T21:25:44.400000Z | 151.6 | -5.67 | 42.2 | 6.9
2018-04-02T13:40:41.300000Z | -62.81 | -20.63 | 569.1 | 6.83
2018-05-04T22:33:10.600000Z | -154.76 | 19.12 | 12.0 | 6.88
2018-08-05T11:46:44.700000Z | 116.24 | -8.33 | 17.8 | 6.94
2018-08-19T00:19:59.000000Z | -177.85 | -17.86 | 555.0 | 8.2
2018-08-19T14:56:35.600000Z | 116.75 | -8.4 | 23.5 | 6.93
2018-08-24T09:04:10.200000Z | -70.82 | -11.18 | 627.7 | 7.09
2018-08-29T03:52:23.600000Z | 169.82 | -22.29 | 17.7 | 6.9
2018-10-10T20:48:31.100000Z | 151.2 | -5.98 | 39.4 | 6.99
2018-11-30T17:29:34.700000Z | -150.02 | 61.49 | 48.2 | 7.05
2018-12-05T04:18:32.700000Z | 169.25 | -21.95 | 17.8 | 7.53
2019-01-05T19:25:41.200000Z | -71.68 | -8.1 | 588.1 | 6.77
2019-02-22T10:17:28.000000Z | -77.09 | -2.26 | 121.1 | 7.48
2019-03-01T08:50:49.000000Z | -70.14 | -14.78 | 273.6 | 7.02
2019-05-06T21:19:44.700000Z | 146.49 | -7.06 | 128.0 | 7.13
2019-05-26T07:41:52.800000Z | -75.36 | -5.25 | 126.6 | 7.99
2019-06-15T22:55:12.500000Z | -177.79 | -30.93 | 47.6 | 7.29
2019-08-01T18:28:16.400000Z | -72.24 | -34.32 | 25.0 | 6.78
2019-11-14T16:17:47.500000Z | 126.32 | 1.66 | 29.6 | 7.09
