# Supplementary material for: An estimate of absolute shear-wave speed in the Earth’s inner core
Source: Nat Commun. 2023 Jul 29;14:4577. doi: 10.1038/s41467-023-40307-9 (PMC10387060; doi:10.1038/s41467-023-40307-9)
Supplement: Supplementary file 3 — Description of Additional Supplementary Files [file 41467_2023_40307_MOESM3_ESM.pdf]

Description of Additional Supplementary Files

Supplementary Movie 1: A series of synthetic global correlograms featuring the detection of several J-wave sensitive correlation features. The change in the timing (vertical axis) of some features in the correlogram as we perturb the J-wave speeds relative to PREM is the evidence for their sensitivities. Identified correlation features sensitive to J-wave speeds are highlighted by insets (black rectangles) in the main correlogram (panel to the left), which are enlarged in panels on the right.
